# Early opportunities for prevention: infections of pregnant women and young infants.

**DOI:** 10.3201/eid0707.017711

**Published:** 2001

**Authors:** A Schuchat, S Hillier, K Edwards, S Schrag, M Labbok

**Affiliations:** Centers for Disease Control and Prevention, Atlanta, Georgia 30333, USA. aschuchat@cdc.gov


### Early Opportunities for Prevention: Infections of Pregnant Women and Young Infants

**Anne Schuchat,* Sharon Hillier,† Kathryn Edwards,‡ Stephanie Schrag,* and Miriam Labbok§**

*Centers for Disease Control and Prevention, Atlanta, Georgia, USA; †University of Pittsburgh-Magee Women's Hospital, Pittsburgh, Pennsylvania, USA; ‡Vanderbilt University School of Medicine, Nashville, Tennessee, USA; and §United States Agency for International Development, Washington, DC, USA

Infectious agents are a leading cause of pregnancy complications and contribute to serious illness, death, and disability in infants. Substantial prevention opportunities exist.

### Role of Infectious Agents in Adverse Consequences of Pregnancy

A variety of studies have assessed the role of sexually transmitted infections (e.g., *Neisseria gonorrhoeae*, *Chlamydia trachomatis*, *Trichomonas vaginalis*) and other genital tract infections (e.g., those caused by group B streptococcus [GBS], bacterial vaginosis [BV]) in preterm deliveries. Although numerous studies have shown that the presence of each of these agents can increase the risk of preterm delivery, nearly every antimicrobial treatment trial failed to show a beneficial effect in reducing preterm deliveries or numbers of newborns of low birth weight. A consistently strong association between BV and preterm delivery has been identified, yet only two randomized controlled trials, out of approximately 15 clinical trials, found that antimicrobial treatment during pregnancy had a positive impact. The typical intervention trial targeted a single infectious agent, even though the agents used have broad effects on multiple organisms and interactions exist among genital tract flora. Future research approaches may benefit from abandoning the paradigm of the randomized controlled trial that attempts to vary a single parameter and instead adopting holistic approaches to address multiple pathogens in concert.

### Maternal Immunization

Vaccination of pregnant women has a long history, and several vaccines are now routinely recommended during pregnancy. An essential factor to consider is that the placenta is an extremely complex immunologic organ, with highly selective placental transport of immunoglobulin G that is receptor-mediated and active. Vaccinations are given in the last trimester to avoid effects on organogenesis. Excellent safety profiles were observed for several vaccines given during a large perinatal project conducted by the National Institutes of Health from 1957 to 1966. Nevertheless, legal liability for manufacturers remains a barrier. The high background rate of congenital anomalies and fetal loss is compounded by the challenges of distinguishing temporal relationships from causal ones. Despite these factors, active research is proceeding on maternal immunization against GBS, *Streptococcus pneumoniae*, and respiratory syncytial virus (RSV) using polysaccharide and protein conjugate vaccines as well as RSV-subunit vaccines (PFP-2).

### Control of Infectious Diseases Through Breast-Feeding

Breast-feeding reduces illness and death from infectious diseases in all socioeconomic settings. Maternal exposure and antibodies generally negate any impact of passage of infectious agents. The one rare, but important, exception is passage of retroviruses, including HIV, in cells. Breast milk contains cellular (e.g., T and B lymphocytes, neutrophils, macrophages) and noncellular (e.g., immunoglobulins, hormones, enzymes) components, both of which contribute to preventing infection. Exclusive breast-feeding is associated with the greatest reduction in illness and death from infectious agents when compared to results from partial breast-feeding. Models have estimated tremendous economic and health benefits of increasing the proportion of women who breast-feed; increases will only occur where breast-feeding skills are supported by both the health and social systems. Studies need to differentiate between exclusive breast-feeding, partial breast-feeding, and exclusive formula feeding to carefully determine risk-benefits for women and infants in particular settings.

### Prevention Success Stories

Several features differentiate perinatal infections from other infectious disease syndromes: 1) the time during which disease transmission can occur is limited; 2) eradication of the pathogen from the mother is not essential in preventing transmission; 3) researchers have avoided testing in pregnant women and newborns because they are vulnerable populations; and 4) pregnancy provides opportunities to integrate public health response. During the 1990s, efforts to prevent perinatal transmission of HIV and GBS in the United States were highly successful, with an 86% reduction in perinatally acquired AIDS and a 70% reduction in early-onset GBS disease. Racial disparity was reduced significantly in both circumstances. Keys to success included advocacy, surveillance, support by professional organizations, and the integration of perinatal prevention programs.

Address for correspondence: Anne Schuchat, Division of Bacterial and Mycotic Diseases, Centers for Disease Control and Prevention, 1600 Clifton Road, Mailstop C23, Atlanta, GA 30333, USA; fax: 404-639-3970; e-mail: aschuchat@cdc.gov

